# Reduced Accumulation Rate and Morphological Changes of Newly Generated Myelinating Oligodendrocytes in the Corpus Callosum of Aged Mice

**DOI:** 10.1002/glia.70070

**Published:** 2025-08-01

**Authors:** Sasikarn Looprasertkul, Reiji Yamazaki, Yasuyuki Osanai, Nobuhiko Ohno

**Affiliations:** ^1^ Department of Anatomy, Division of Histology and Cell Biology, School of Medicine Jichi Medical University Shimotsuke Japan; ^2^ Division of Ultrastructural Research National Institute for Physiological Sciences Okazaki Japan

**Keywords:** aging, corpus callosum, myelination, newly generated oligodendrocyte, oligodendrocyte

## Abstract

The activity of oligodendrocyte progenitor cells (OPCs) and oligodendrocytes (OLs) throughout life drives myelination, which is crucial for rapid neuronal communication. OLs in the aging brain demonstrate a reduced capacity for myelin formation and maintenance, but the underlying differentiation of individual OLs and morphological changes of their myelin in aging remain unclear. Here, we utilized *Pdgfra‐CreER* ^*T2*^:*Tau‐mGFP* double transgenic mice to selectively label and visualize newly generated OLs in aged (78‐week‐old) mice and compared them with those in young (8‐week‐old) mice. We revealed a significantly lower percentage of newly generated OLs that differentiated into mature OLs and a decreased rate of myelinating OLs accumulation in aged mice compared with young mice. Additionally, newly generated myelinating mature OLs in aged mice demonstrated significantly greater height compared with those in young mice. Furthermore, myelin internodes were significantly shorter and significantly fewer in aged mice compared with young mice. Our results indicate age‐related impairments in the differentiation efficiency of aged OPCs and age‐related morphological changes in OLs. These alterations in newly generated OLs may contribute to impaired myelination, reduced myelin turnover, and disrupted myelin maintenance in aged mice.

## Main Points

- Newly generated myelinating oligodendrocyte accumulation is decreased in the corpus callosum of aged mice.
- Morphological changes of newly generated myelinating oligodendrocytes in aged mice: greater height, shorter and fewer myelin sheaths.

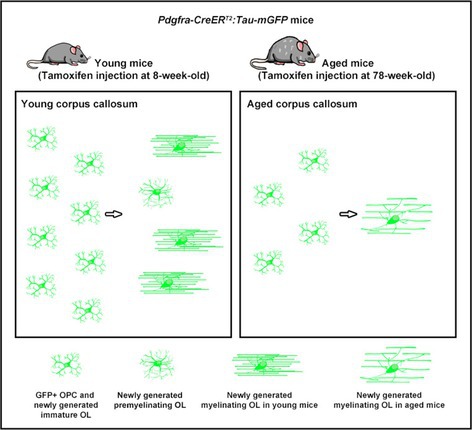

## 1 Introduction

Oligodendrocytes (OLs), which are cells responsible for myelination in the central nervous system (CNS), are generated during development from OL progenitor cells (OPCs) (Bercury and Macklin 2015). New OLs extend processes and rapidly establish distinct ensheathed axonal segments, called internodes (Osso and Hughes 2024), between the nodes of Ranvier (Fields 2014) by wrapping their multi‐layered lipid membranes around axons (Mathews and Appel 2016). The internodal myelin sheaths are anchored to the axolemma at the paranode, located at the tips of each internode, through specialized proteins, including Caspr and contactin (Boyle et al. 2001; Rios et al. 2000). Multiple structural factors of myelin, such as the length of myelin internodes and nodes and myelin thickness, affect the speed of nerve conduction (Fields 2014). The structures of mature myelin are dynamic, and it undergoes both membrane renewal and remodeling for myelin turnover, which is crucial for maintaining rapid neuronal communication (Osso and Hughes 2024). Myelin renewal and remodeling occur throughout life via both preexisting OLs and newly generated OLs (Osso and Hughes 2024; Sampaio‐Baptista and Johansen‐Berg 2017). A considerable amount is now known about the effects of aging on adult OPCs and myelin regeneration.

Aging causes various functional and morphological changes in the CNS, including cerebral atrophy (Lasiene et al. 2009). These changes in aging are primarily characterized by both gray and white matter volume reductions, ventricular enlargement, and gyrification loss (Fjell et al. 2014). A decline in nerve conduction efficiency, including decreased conduction amplitudes, prolonged latencies, and reduced conduction velocity, is observed as aging progresses (Shelly et al. 2023). The decline in nerve conduction could be attributable to the reduced myelin formation, maintenance, and regeneration capacity of OLs in the aging brain (Sams 2021). Additionally, aging is associated with myelin degeneration and axonal internode loss, which may contribute to cognitive decline and the development of several neurological disorders (Hill et al. 2018). The proteomic analysis of OPCs shows another evidence of impaired OL functions in aging, revealing age‐related alterations in cholesterol biosynthesis and protein homeostasis pathways (de la Fuente et al. 2020). Consistently, both OL differentiation and remyelination efficiency significantly decline in older rodents after toxin‐induced demyelination (Cantuti‐Castelvetri et al. 2018; Sim et al. 2002). Active myelin renewal and/or remodeling would be crucial for preserving proper brain function and preventing age‐related cognitive impairment, considering these changes in OLs, myelin, and brain functions observed in aging. However, the capacity of OPCs that differentiate into newly generated mature OLs and that contribute to myelin formation and maintenance remains unclear in the context of CNS aging.

The corpus callosum (CC) is a major white matter tract in the brain that connects the left and right hemispheres and is crucial for transmitting motor, sensory, and cognitive information between them (Roland et al. 2017). Myelinated axons in the mouse CC first appeared on postnatal day 11, and their percentage steadily increased from postnatal day 14 to 45 (Sturrock 1980). Approximately 90% of myelinating OLs generated during a young age persisted until 20 months of age in mice (Tripathi et al. 2017). Conversely, myelination rates significantly decline after 90 days postnatal (Sturrock 1980), indicating impaired oligodendrogenesis and myelin formation in the CC in aging. However, the accumulation rate and the structural characteristics of newly generated OLs in the aged CC remain unknown.

In this study, we investigated the oligodendrogenesis and morphology of newly generated OLs after aging in the CC to address these questions. We used *Pdgfra‐CreER* ^*T2*^:*Tau‐mGFP* double transgenic mice to specifically label newly generated OLs after tamoxifen injection in both young (8‐week‐old) and aged (78‐week‐old) mice and to trace their differentiation into mature OLs over 2 or 4 weeks. We successfully identified individual newly generated mature OLs that differentiated from OPCs in both young and aged mice in the CC. Our results indicate that oligodendrogenesis and the rate of myelinating OLs accumulation decreased and that the morphology of the newly generated OLs changed with the aging process. Additionally, distinct morphological differences in newly generated OLs in aged mice may be involved in impaired myelination and myelin maintenance disruption after aging.

## 2 Materials and Methods

### 2.1 Animals

Japan SLC (Shizuoka, Japan) supplied C57BL/6 mice. Dr. William Richardson (Rivers et al. 2008) gifted *Pdgfra‐CreER* ^*T2*^ mice, and the Jackson Laboratory (JAX stock # 021162) provided *Tau‐mGFP* reporter mice (Hippenmeyer et al. 2005). *Pdgfra‐CreER* ^*T2*^ (either homozygous or heterozygous) and *Tau‐mGFP* (heterozygous) mice were generated by the crossbreeding of *Pdgfra‐CreER* ^*T2*^ transgenic mice with *Tau‐mGFP* homozygous mice. The Institutional Animal Care and Use Committee approved all experiments, which were conducted under the guidelines for the care and use of animals at Jichi Medical University (approval no. 23005‐05) and the ARRIVE (Animal Research: Reporting of In Vivo Experiments) guidelines. The animals were housed in transparent cages (CL‐0104‐3; 207 × 314 × 128 mm; CLEA Japan Inc.) under standard laboratory conditions at room temperature (RT; 20°C–25°C) with a 12‐h light/dark cycle (7 a.m.–7 p.m.) and constant access to food and water. All animals were housed in pairs or groups of up to five animals per cage. This study used 24 mice, which included 11 males and 13 females. The numbers of males and females in each of the 8 + 2 W, 78 + 2 W, and 78 + 4 W groups were as follows: eight mice including three males and five females in the 8 + 2 W group, eight mice including five males and three females in the 78 + 2 W group, and eight mice including three males and five females in the 78 + 4 W group.

### 2.2 Genotyping

All pups (P8–P10) were genotyped using PCR assays. Total DNA was extracted from digit biopsies by incubation in 90 μL 50 mM NaOH at 95°C for 10 min, followed by the addition of 110 μL 100 mM Tris–HCl (pH 7.6). The mixture was then centrifuged at 12,000 rpm (12,477×*g*) in a high‐speed centrifuge (15.5M‐18AL angle rotor; M201‐IVD, Sakuma, Tokyo, Japan) at 4°C for 3 min. Conventional multiplex PCR was then conducted using reagents from PCR master mix KOD FX Neo (TOYOBO, Osaka, Japan) in 10‐μL final volumes and the GeneAmp PCR System 9700 thermal cycler (Applied Biosystems, USA). The Cre‐recombinase expression was examined with forward (5′‐CAGGTCTCAGGAGCTATGTCCAATTTACTGAACGTA‐3′) and reverse (5′‐GGTGTTATAAGCAATCCCCAGAA‐3′) primers, yielding a product size of 525 base pairs. *Tau‐mGFP* was investigated with a forward primer (5′‐CCTTGTCCCCAACTCCATAC‐3′), a reverse wild‐type primer (5′‐TGTGTATGTCCACCCCACTG‐3′), and a reverse mutant primer (5′‐AAACATGTCCCAGCTCCAAG‐3′), with product sizes of 224 and 280 base pairs, respectively.

### 2.3 Tamoxifen Administration

Tamoxifen (Nacalai Tesque, Kyoto, Japan) was dissolved in corn oil (Sigma, St. Louis, USA) to a 10‐mg/mL final concentration. *Pdgfra‐CreER* ^*T2*^:*Tau‐mGFP* double transgenic mice were intraperitoneally injected with tamoxifen at 100 mg/kg for four consecutive days at 8 or 78 weeks of age.

### 2.4 Immunohistochemistry and Imaging

The *Pdgfra‐CreER* ^*T2*^:*Tau‐mGFP* double transgenic mice were intraperitoneally injected with the anesthetic three drugs mixture (medetomidine of 0.3 mg/kg, midazolam of 4.0 mg/kg, and butorphanol of 5.0 mg/kg) and transcardially perfused with 4% paraformaldehyde (PFA) in phosphate buffer (PB) of 0.1 M at a pH of 7.4 at 10 weeks (2 weeks after tamoxifen injection at 8 weeks old), 80 weeks (2 weeks after tamoxifen injection at 78 weeks old), or 82 weeks of age (4 weeks after tamoxifen injection at 78 weeks old). The brains were post‐fixed in the buffered 4% PFA overnight at 4°C and coronally sliced with a vibratome slicer (LinearSlicer PRO 10; DSK, Kyoto, Japan) to obtain 100 μm‐thickness brain slices. The brain slices were separated into three areas, including anterior (bregma 0.37 to 0.73), middle (bregma −0.47 to −0.83), and posterior (bregma −1.67 to −2.03). Additionally, immunostaining was performed following the RapiClear 1.52 protocol (RC152001, Sunjin Laboratory, Taiwan, China) with minor modifications. In brief, the floating brain slices were placed in a 24‐well plate and washed thrice for 15 min each with phosphate‐buffered saline (PBS) at RT and incubated in 2% PBST (2% Triton X‐100 in PBS solution containing 0.05% sodium azide) on an orbital shaker for 1 day at RT for permeabilization. After 1 day, the brain slices were washed thrice with PBS for 15 min each at RT and incubated in freshly prepared blocking buffer with Mouse on Mouse (M.O.M.) Blocking Reagent (one drop of M.O.M. (Vector Laboratories, California, USA) in 1 mL of 10% normal goat serum, 1% Triton X‐100, 2.5% DMSO, and 0.2% sodium azide in PBS) on an orbital shaker at 4°C for 1 day. Slices were then incubated in antibody dilution buffer (1% normal goat serum, 0.2% Triton X‐100, 2.5% DMSO and 0.1% sodium azide in PBS) on an orbital shaker at 4°C for 3 days with the following primary antibodies: monoclonal rat anti‐green fluorescent protein (GFP, 1:500, IgG2a, 04404‐84, Nacalai Tesque), monoclonal mouse anti‐adenomatous polyposis coli (APC or CC1, 1:100, IgG2b, OP80, Merck, Darmstadt, Germany), polyclonal rabbit anti‐human Olig2 (1:100, 18953, Immuno‐Biological Laboratories America, Minneapolis, USA), polyclonal rabbit anti‐contactin‐associated protein 1 (Caspr, 1:1000, ab34151, Abcam, Massachusetts, USA), monoclonal mouse anti‐glial fibrillary acidic protein (GFAP, 1:100, IgG1, G3893, Sigma), and polyclonal rabbit anti‐ionized calcium‐binding adapter molecule 1 (Iba1, 1:100, 019‐19741, Wako, Osaka, Japan), polyclonal rabbit anti‐NG2 chondroitin sulfate proteoglycan (NG2, 1:50, AB5320, Merck). The brain slices were washed thrice with washing buffer for 1 h each at RT and incubated in washing buffer (3% NaCl and 0.2% Triton X‐100 in PBS) at 4°C overnight. Secondary antibodies in antibody dilution buffer, including goat anti‐rat IgG conjugated with Alexa Fluor 488 (1:250, A‐11006, Thermo Fisher, Massachusetts, USA), goat anti‐rabbit IgG conjugated with Alexa Fluor 568 (1:250, A‐11011, Thermo Fisher), and goat anti‐mouse IgG conjugated with Alexa Fluor 633 (1:250, A‐21052, Thermo Fisher) were used to subsequently incubate the brain slices for 2 days at 4°C. They were washed with a washing buffer thrice for 1 h each at RT and then overnight at 4°C. After one overnight, the slices were washed in PBS thrice for 15 min each at RT. Thereafter, they were incubated with Hoechst 33342 (1:1500, H3570, Thermo Fisher) for nuclear labeling for 2 h at RT. After a final wash with PBS thrice for 15 min each at RT, the slices were placed on glass slides and mounted with RapiClear warming solution (37°C) of 30 μL along with an antifade solution (ProLong Gold Antifade Reagent, Thermo Fisher).

Images were taken from a minimum of six brain slices per mouse (two brain slices per area) after two different types of immunostaining. To identify the region of interest (ROI), montages of single confocal scans were acquired using a Dragonfly high‐speed confocal microscope system with Fusion software (Oxford Instruments, Abingdon‐on‐Thames, UK). A 10× objective lens (N.A. 0.30) was utilized to capture a montage of whole coronal brain slices at a 0.65 μm/pixel resolution with 30% overlapping of one plane. The images were auto‐stitched using Fusion software to produce a single image for analysis. Images for OL counting and individual OL analysis were obtained with a 60× objective lens (N.A. 1.42), capturing a series of z‐stack projections, consisting of 50–200 single planes with a step size of 0.5 μm at a 0.1083 μm/pixel resolution. Images were exported from Fusion software in IMS file format (.ims), which retains the z‐plane information.

### 2.5 Analysis of the Confocal Images

Serial z‐stack images, including the entire depth of individual cells, were acquired, imported into Fiji software, and then exported to TIFF (.tif) or Portable Network Graphics (.png) file formats. To investigate the density of newly generated OLs, imaging was performed within an ROI spanning a width of 4000 μm (ROI extended 2000 μm laterally from the brain midline on each side). To investigate the density of cells, the number of OLs per mouse was counted and determined to be: 40–107 in the 8 + 2 W group, 16–57 in the 78 + 2 W group, and 15–59 in the 78 + 4 W group. To investigate the morphology of individual newly generated myelinating mature OLs, the numbers of imaged OLs in each group were: 3–12 cells per mouse and a total of 50 cells from seven mice in the 8 + 2 W group, and 1–5 cells per mouse and a total of 24 cells from nine mice in the aged (78 + 2 W and 78 + 4 W) group. The GFP^+^ myelinating mature OLs in the 78 + 2 W and the 78 + 4 W groups were combined for the morphological analyses because the number of GFP^+^ myelinating mature OLs in aged mice was much lower than that in young mice, and there was no discernible morphological difference between OLs in the 78 + 2 W and 78 + 4 W groups. All Olig2^+^GFP^+^ cells within 100 μm‐thick coronal sections of the anterior, middle, and posterior CC (located within 4000 μm including 2000 μm lateral to each side of the brain midline) were counted. The size of the analyzed CC region in each slice was then measured with Fiji, and the cell numbers were normalized to 1 mm^2^ to give a cell density. Morphological analysis of z‐series images of individual newly generated myelinating mature OLs determined their height by identifying the points where processes connect to the outermost dorsal and ventral internodes. Then, compressed confocal stack images were used to measure the linear distance between the outermost dorsal and ventral internodes, which represented the “height” of the cell. Myelin internodes were manually traced as the length of myelin processes in z‐stack series images using the Simple Neurite Tracer (SNT) reconstruction plugin in Fiji (Arshadi et al. 2021). The presence of the paranode marker Caspr or the characteristic morphology of myelin tips (larger size and dense fluorescence intensity) was used to define internode boundaries (Ford et al. 2015; Osanai et al. 2022). The analysis excluded internodes that lacked two identifiable myelin tips. Additionally, the number of myelin internodes was counted with SNT reconstruction of z‐stack series images. Fiber diameter measurements were performed according to a previous study with minor modifications (Tanaka et al. 2021), as described in Methods S1.

### 2.6 Statistical Analysis

GraphPad Prism and R studio (R Core Team 2025) software were used for statistical analyses. Data were tested for normality with a Shapiro–Wilk test by GraphPad Prism. Data that were normally distributed were analyzed using a parametric test, Student's *t*‐test for two groups comparison and one‐way analysis of variance (ANOVA) for three group comparisons, followed by a Tukey's multiple comparisons test by GraphPad Prism, and these data are expressed as the mean ± standard error of the mean (SEM). When data were not meet normally distributed, nonparametric Mann–Whitney *U*‐test for two‐group comparisons by GraphPad Prism and a Scheirer–Ray–Hare test for three group comparisons, followed by a Dunn's multiple comparisons test by R studio, were used. These data are expressed as the median (interquartile range (IQR)). Pearson's correlation coefficient was used to compare the correlation between two groups by GraphPad Prism. The Kolmogorov–Smirnov test was employed to compare the cumulative distribution of data by GraphPad Prism. Statistical significance is indicated by asterisks; *p < 0.05, **p < 0.01, ***p < 0.001, ****p < 0.0001.

## 3 Results

### 3.1 *Pdgfra‐CreER^T2^:Tau‐mGFP* Double Transgenic Mice Enable Specific Labeling of Newly Generated OLs

**FIGURE 1:**
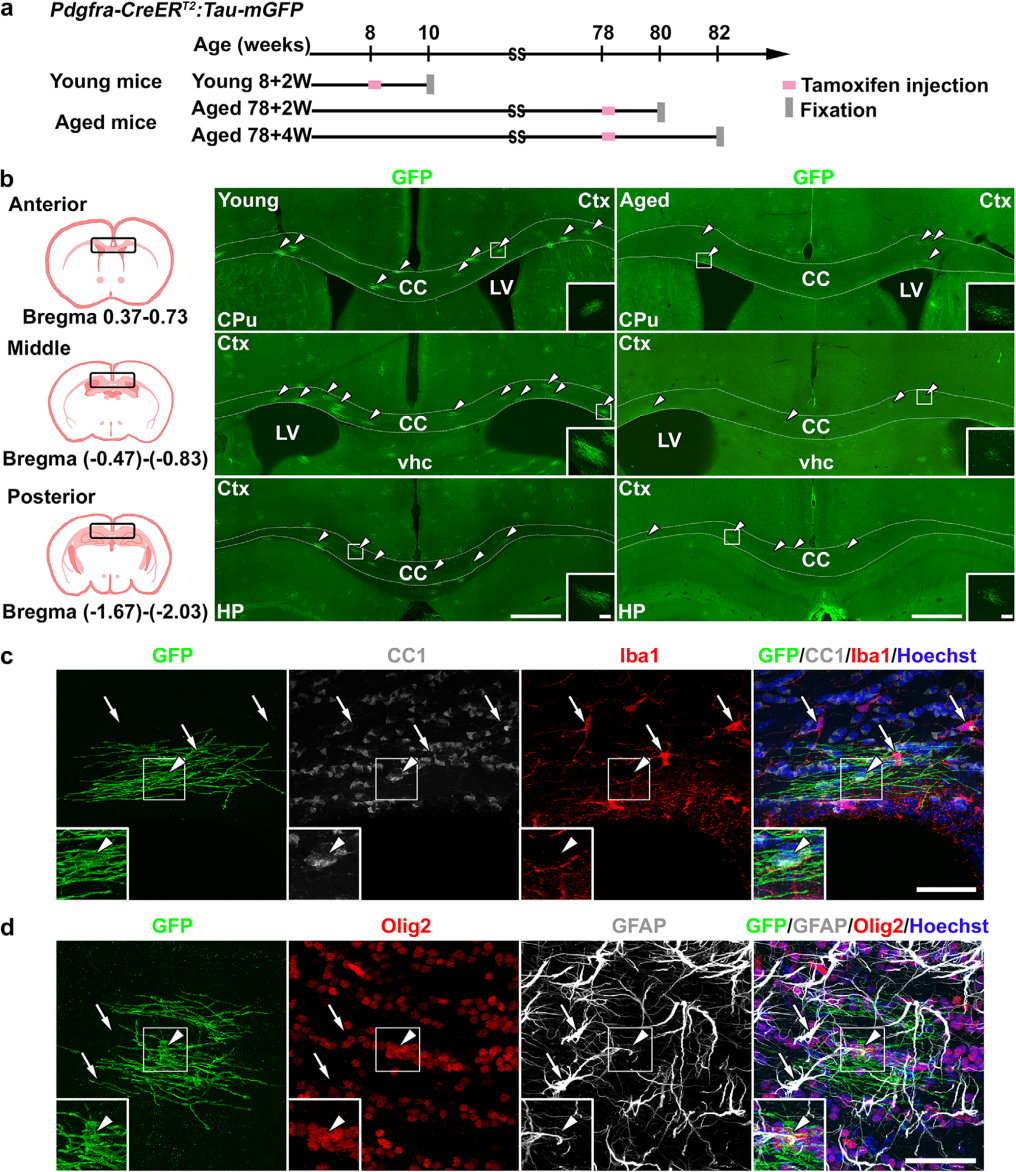
Newly generated oligodendrocytes labeled in *Pdgfra‐CreER* ^*T2*^:*Tau‐mGFP* mice. (a) A scheme illustrating the experimental protocol. *Pdgfra‐CreER* ^*T2*^:*Tau‐mGFP* double transgenic mice were categorized into two groups according to the tamoxifen injection time point: 8‐week‐old (young) and 78‐week‐old (aged). Young mice were fixed 2 weeks following the tamoxifen injection (8 + 2 weeks). Aged mice were fixed at two intervals: 2 weeks (78 + 2 weeks) and 4 weeks (78 + 4 weeks) following the tamoxifen injection. (b) Schemes of coronal brain slices (left panels) and representative fluorescent images of GFP‐positive cells (arrowheads) in the slices from the young (middle panels) and aged (right panels) transgenic mice at low and high magnifications. The rectangles in the middle and right panels are magnified in the insets. The slices were collected from three different levels in the rostral‐caudal axis: Anterior (bregma 0.37 to 0.73), middle (bregma −0.47 to −0.83), and posterior (bregma −1.67 to −2.03). The schemes demonstrate the observed areas (black rectangles, left panels). Dashed lines demarcate the corpus callosum (CC). Cpu, caudate putamen; Ctx, cortex; HP, hippocampus; LV, lateral ventricle; vhc, ventral hippocampal commissure. Scale bars: 500 μm (low magnification) and 50 μm (high magnification, in the insets). (c, d) Immunofluorescence staining for GFP (c, green), CC1 (c, white), and Iba1 (c, red) or GFP (d, green), Olig2 (d, red), and GFAP (d, white) in the CC of aged transgenic mice. GFP‐positive cells, which are magnified in the insets, colocalize with CC1 (c, arrowheads) and Olig2 (d, arrowheads) but not with Iba1 (c, arrows) or GFAP (d, arrows). Nuclei are counterstained with Hoechst (blue). Scale bars: 50 μm.

We utilized a *Pdgfra‐CreER* ^*T2*^:*Tau‐mGFP* reporter mouse line to label newly generated OLs after tamoxifen‐induced Cre recombination and to investigate changes in the accumulation of mature OLs in the aged brain. In this mouse line, expression of mGFP in differentiated OLs is controlled by the *Tau* promoter, and mGFP was observed only in OLs that underwent Cre recombination by tamoxifen administration when they were *Pdgfra*‐positive OPCs. Tamoxifen was administered to both young (8‐week‐old) and aged mice (78‐week‐old) for four consecutive days, and we fixed and immunostained the brain tissues and observed the presence of GFP‐positive cells in the CC of all three groups after a 2‐week (8 + 2 W, 78 + 2 W) or 4‐week period (78 + 4 W) (Figure 1a). Immunostaining for GFP clearly visualized GFP‐positive cells in the CC located within 4000 μm from the CC center, with or without classifying the anterior, middle, and posterior CC areas in each mouse group. The immunostaining revealed many GFP‐positive cells in the CC of young mice, and the density of GFP‐positive cells appeared lower in aged mice than in young mice (Figure 1b). In contrast, no GFP‐positive cells without tamoxifen injection were observed (Figure S1). Immunostaining for GFP along with CC1 (mature OL marker), Olig2 (OL lineage marker), Iba1 (microglia marker), and GFAP (astrocyte marker) demonstrated that GFP immunoreactivity colocalized with an Olig2‐positive and CC1‐positive OL fraction, but not with GFAP‐positive astrocytes and Iba1‐positive microglia (Figure 1c,d, Methods S1, Table S1, and Figure S2). These results indicated that we successfully labeled the OLs newly generated after tamoxifen injection.

### 3.2 Accumulation of Myelinating OLs Is Reduced in the CC of Aged Mice

**FIGURE 2:**
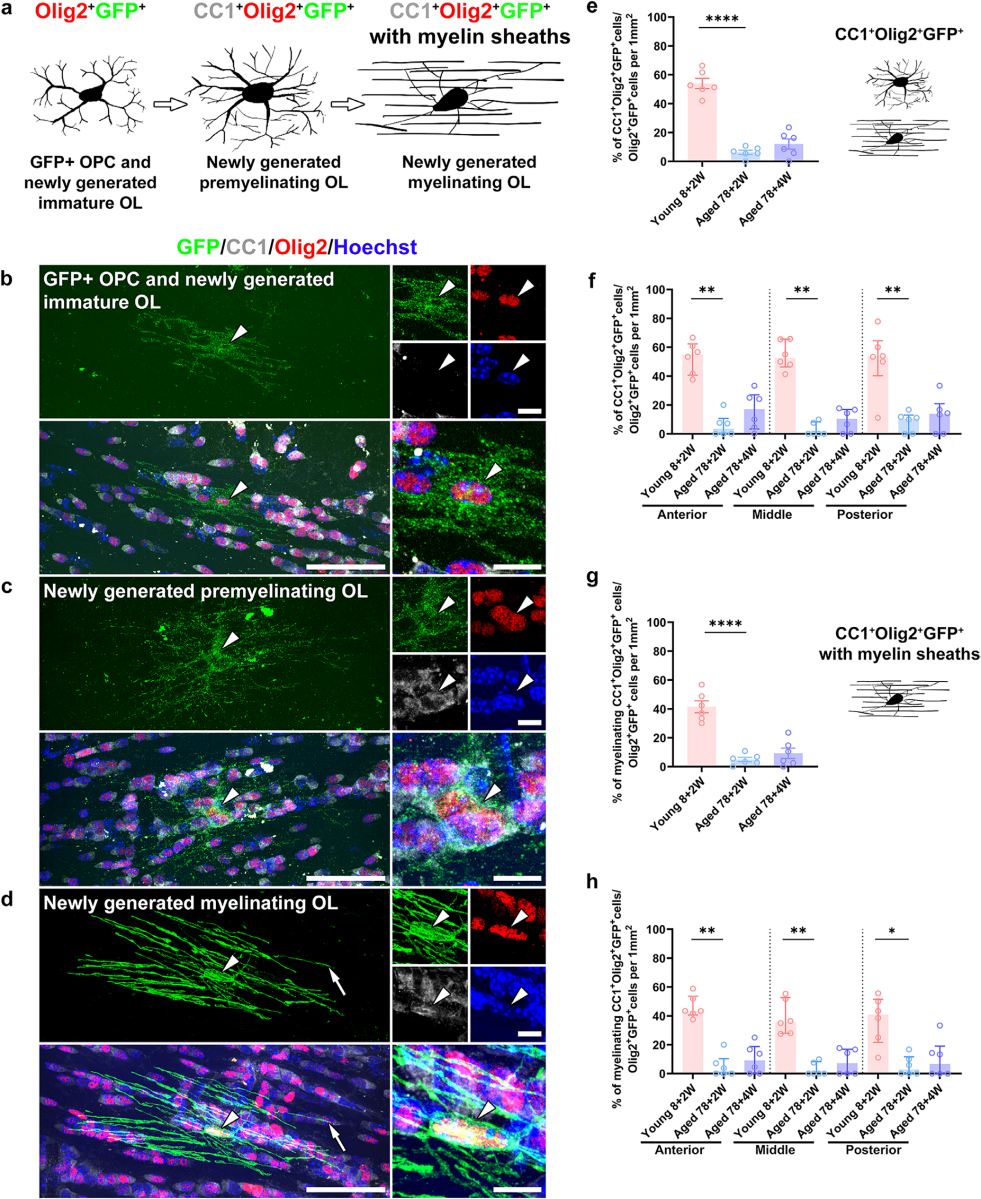
Impaired accumulation rate of newly generated myelinating mature oligodendrocytes in aged mice. (a) Diagram of the three different types of GFP‐positive cells in *Pdgfra‐CreER* ^*T2*^:*Tau‐mGFP* transgenic mice. GFP^+^ oligodendrocyte progenitor cells (OPCs) and newly generated immature oligodendrocytes (OLs) positive for GFP and Olig2 (left) differentiate into newly generated premyelinating mature OLs positive for GFP, Olig2, and CC1 (middle), which then differentiate into newly generated myelinating mature OLs positive for GFP, Olig2, and CC1 with myelin sheaths (right). (b–d) GFP (green), Olig2 (red), and CC1 (white) immunostaining in the corpus callosum (CC) from aged transgenic mice. One of the GFP^+^ OPCs and newly generated immature OLs is positive for GFP and Olig2 but negative for CC1 (b, arrowheads). The newly generated premyelinating mature OL (c, arrowheads) and newly generated myelinating mature OL (d, arrowheads) are immunopositive for GFP, Olig2, and CC1, with the latter having straight GFP‐positive processes corresponding to myelin internodes (d, arrows). Nuclei are counterstained with Hoechst (blue). Scale bars: 50 (left) and 10 (right) μm. (e, f) The proportion of newly generated premyelinating and myelinating mature OLs in the newly generated OLs in the entire CC (e) or the anterior, middle, and posterior CC parts (f) in the three groups. (g, h) The proportion of newly generated myelinating mature OLs in the entire CC (g) or the anterior, middle, and posterior CC parts (h) in the three groups. *N* = 6 mice per group; three males and three females; three coronal slices per mouse; one coronal slice per area in each mouse (f, h) or three coronal slices from each mouse were pooled (e, g). Data are expressed as mean ± SEM (e, g) and median (IQR) (f, h). One‐way ANOVA (e, g) or Scheirer–Ray–Hare test (f, h). *p < 0.05; **p < 0.01, ****p < 0.0001.

OL lineage cells were classified into morphologically distinct stages during differentiation (Ulloa‐Navas et al. 2021). The GFP‐positive cells were categorized into three distinct groups following the morphology visualized by GFP immunostaining and the markers of OL lineage cells to compare the differentiation stages of OLs labeled with GFP in the *Pdgfra‐CreER* ^*T2*^:*Tau‐mGFP* mice between young and aged mice (Figure 2a). The groups of GFP‐positive cells include GFP^+^ OPCs and newly generated immature OLs positive for GFP and Olig2 but not for CC1, newly generated premyelinating mature OLs positive for GFP, Olig2, and CC1, and newly generated myelinating mature OLs positive for GFP, Olig2, and CC1 with myelin sheath profiles (Figure 2b–d, Figure S3a–c,4a,b). The OL density in the CC at 78 + 2 weeks (12.6 ± 2.9 OLs per mm^2^) was significantly lower than that at 8 + 2 weeks (33.5 ± 4.5 OLs per mm^2^, *p* = 0.0013) when we investigated the density of all the GFP‐positive OLs, which were positive for GFP and Olig2, in mice 2 weeks after tamoxifen injection (Figure S3d). A subset of recombined OPCs is labeled with GFP in *Tau‐mGFP* reporter mice (Pitman et al. 2020), and the proportion of recombined OPCs (NG2^+^GFP^+^ cells) in total GFP^+^ cells or total NG2^+^ cells was not significantly different between young and aged mice (Figure S4). We investigated the proportion of newly generated premyelinating and myelinating mature OLs, which were positive for GFP, Olig2, and CC1, among all the GFP‐positive OLs to further investigate the accumulation of these newly generated OLs. The percentage of newly generated mature OLs in 78 + 2 weeks (6.2% ± 1.5%) was significantly lower than that in 8 + 2 weeks (54.0% ± 3.5%, *p* < 0.0001) (Figure 2e). Additionally, the percentage of newly generated myelinating mature OLs in all the newly generated OLs in 78 + 2 weeks (4.9% ± 1.5%) was significantly lower than that in 8 + 2 weeks (41.4% ± 4.1%, *p* < 0.0001) (Figure 2g). We compared the proportion of all the newly generated OLs in the CC of aged mice 2 (78 + 2 weeks) and 4 (78 + 4 weeks) weeks after tamoxifen injection to assess whether extending the post‐injection period influenced the accumulation rate of newly generated myelinating OLs that differentiate from immature OLs. We revealed no significant difference in the density of newly generated OLs between 78 + 2 weeks and 78 + 4 weeks (10.9 ± 2.2 OLs per mm^2^) (Figure S3d). Additionally, no significant difference in the percentage of mature OLs and myelinating mature OLs was observed between 78 + 2 weeks and 78 + 4 weeks (12.0% ± 3.6% and 9.22% ± 3.6%, respectively) (Figure 2e,g).

We compared the proportions of the groups of OLs in three different CC areas to further investigate whether the accumulation of newly generated mature OLs is different among areas of the CC (anterior, middle, and posterior, Figure 1b). The proportions in all the CC areas at 8 + 2 weeks were higher than those at 78 + 2 weeks, and the proportions in 78 + 2 weeks were not significantly different from those at 78 + 4 weeks (Figure 2f,h); however, there were no significant differences between CC regions within any age group. These results indicated that differentiation of newly generated OLs in aged mice was impaired in all CC areas and was characterized by a lower accumulation rate of myelinating mature OLs.

### 3.3 Age‐Related Changes in the Morphology of Newly Generated Myelinating Mature OLs

**FIGURE 3:**
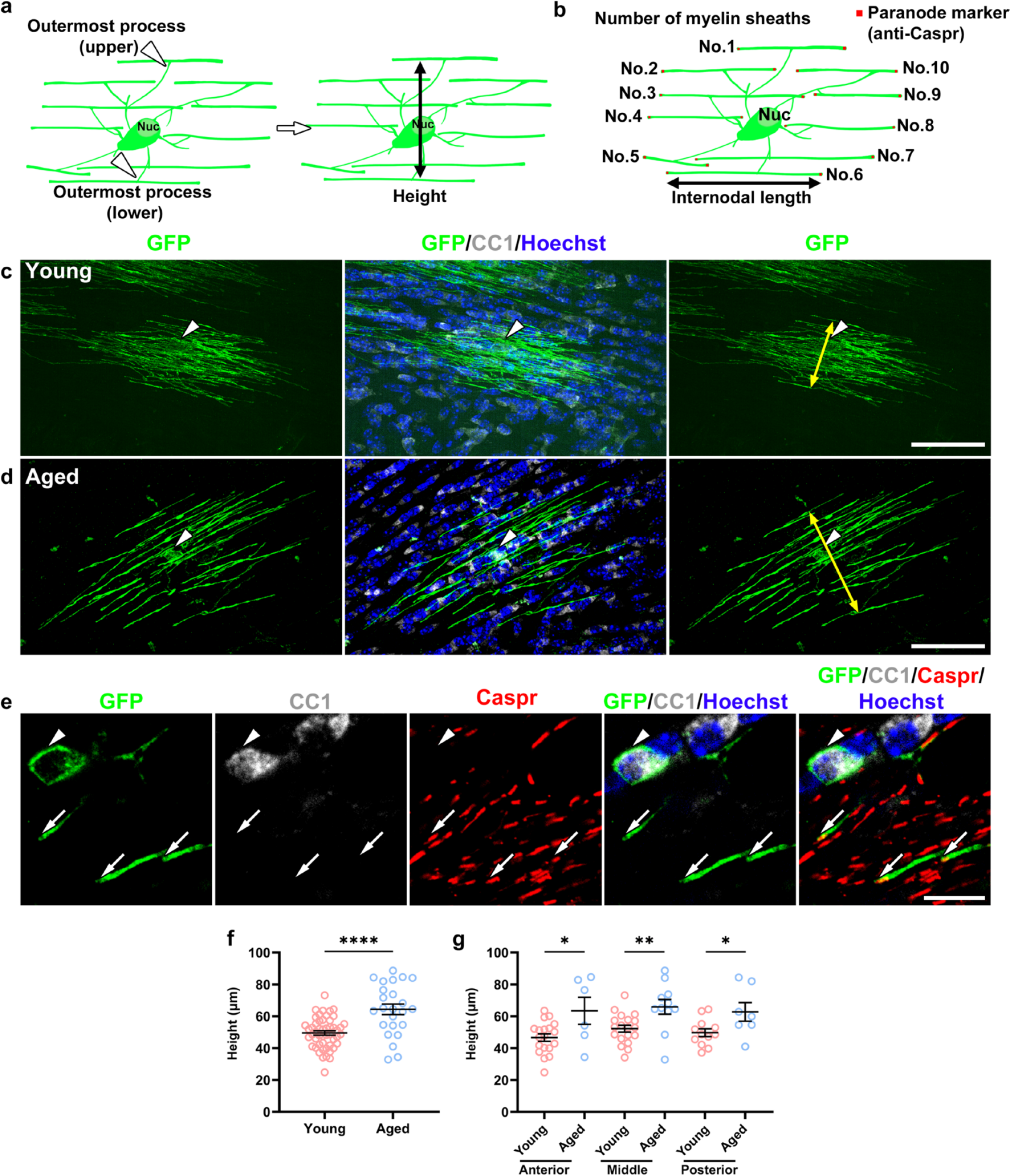
Increased height of newly generated myelinating mature oligodendrocytes in aged mice. (a, b) Schemes illustrating myelinating mature OLs with morphological parameters measured in this study. The height is the distance between the outermost myelin sheaths (a, arrowheads, left) measured over the cell body (a, double‐headed arrow, right). In addition to the number (No.) of myelin sheaths, the internodal length of each myelin sheath is the distance between Caspr immunostaining flanking the internode (b, double‐headed arrow). (c, d) Immunofluorescence staining for GFP (green) and CC1 (white) along with nuclear labeling with Hoechst (blue) demonstrates individual newly generated myelinating mature OLs in young (c, arrowhead) and aged (d, arrowhead) mice. The height in the aged mouse (d, double‐headed arrow) was greater than that in the young mouse (c, double‐headed arrow). Scale bars: 50 μm. (e) Immunostaining for GFP, CC1, and Caspr (red) along with nuclear labeling with Hoechst in the CC of aged mice illustrates the myelin internode of a newly generated myelinating mature OL labeled with GFP (e, arrowhead) flanked by Caspr immunostaining (e, arrows). Scale bar: 10 μm. (f, g) Height of newly generated myelinating mature OLs in young and aged mice in the entire CC (f) or the anterior, middle, and posterior parts of the CC (g). *N* (young, aged) = (50 cells; seven mice, 24 cells; nine mice, f), (anterior: 19 cells, six cells; middle: 19 cells, 11 cells; posterior: 12 cells, seven cells, g). Data are expressed as mean ± SEM. Student's *t*‐test. *p < 0.05, **p < 0.01, ****p < 0.0001.

OL morphology has been significantly affected in aged animals (Young et al. 2013), but it is unclear if the morphology of newly generated OLs and their myelin sheaths as well as their differentiation are affected in aged mice. Hence, we compared the morphology of the newly generated myelinating mature OLs in young (8 + 2 weeks) and aged mice (78 + 2 weeks and 78 + 4 weeks). We defined the height, the number of myelin sheaths, and the internodal length of the individual newly generated myelinating mature OLs to describe the morphology of the newly generated myelinating mature OLs (Figure 3a,b). The height was the distance between the outermost processes of each OL (Figure 3a,c,d), and the tips of the myelin sheaths were confirmed with a paranodal marker, Caspr (Figure 3e). OL height in aged mice was significantly greater than that in young mice in the representative images of newly generated myelinating mature OLs labeled with GFP (Figure 3c,d), which was statistically confirmed (aged: 64.3 ± 3.3 μm vs. young: 49.5 ± 1.4 μm, *p* < 0.0001) (Figure 3f). The difference in height between the young and aged mice was consistent throughout the anterior, middle, and posterior CC areas (Figure 3g). These results indicated that the height increase was a morphological hallmark of the newly generated myelinating OLs during aging.

**FIGURE 4:**
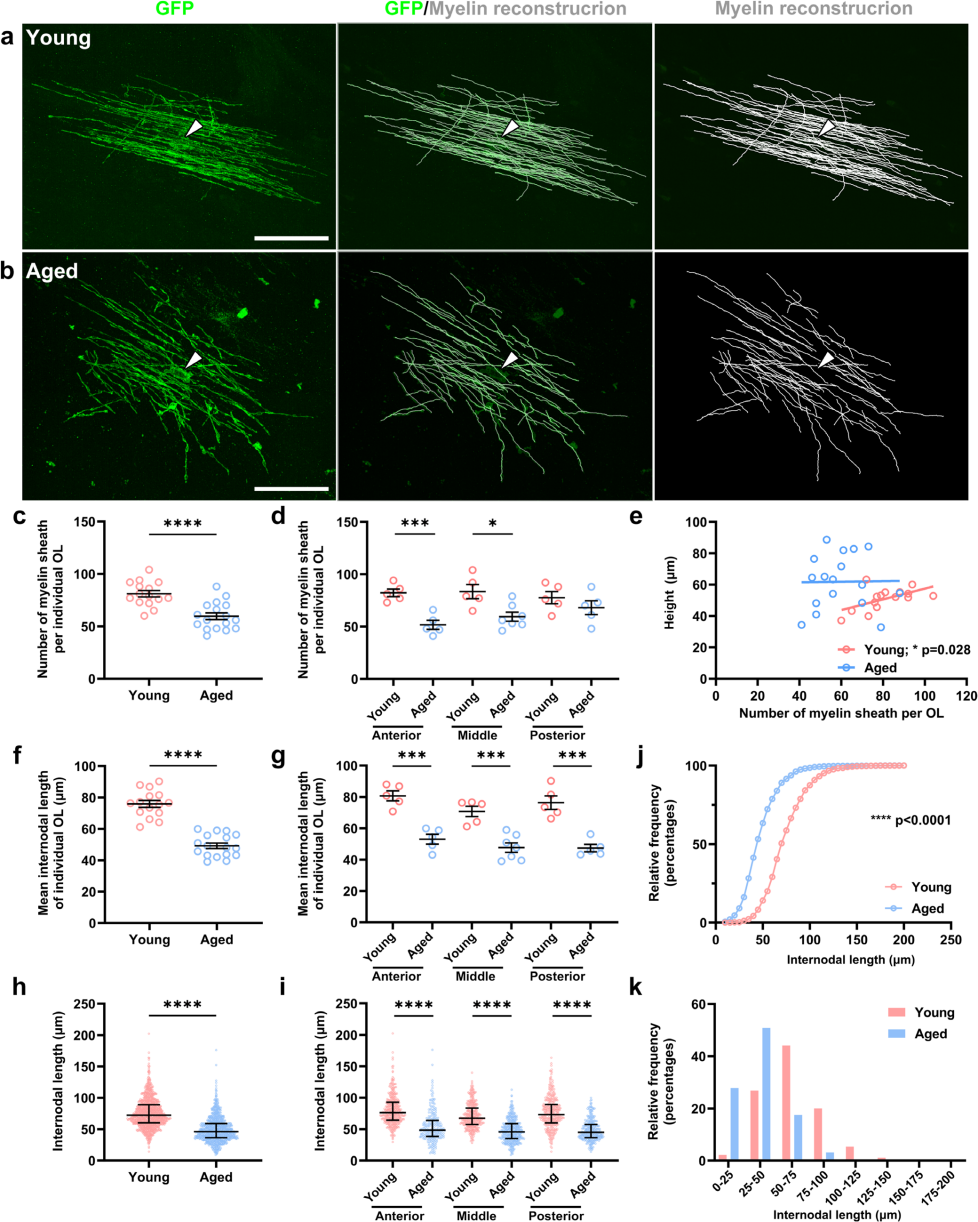
Myelin internodes and myelin sheath numbers of newly generated myelinating mature oligodendrocytes were shorter and fewer, respectively, in aged mice than in young mice. (a, b) Immunofluorescence staining for GFP (green) labeling the newly generated myelinating mature OLs (arrowheads) in the CC of young (a) and aged (b) *Pdgfra‐CreER* ^*T2*^:*Tau‐mGFP* double transgenic mice. White lines (a, b right panels) denote GFP‐positive processes corresponding to myelin sheaths. Scale bar: 50 μm. (c, d) The average number of myelin sheaths per newly generated myelinating mature OLs in young and aged mice in the entire CC (c) or the anterior, middle, and posterior parts of the CC (d). (e) The correlation between the height and the number of sheaths for young (red dots) and aged mice (blue dots). (f, g) The average myelin sheath length of newly generated myelinating mature OLs in young and aged mice in the entire CC (f) or the anterior, middle, and posterior CC parts (g). (h, i) The individual myelin sheath length of newly generated myelinating mature OLs in young and aged mice in the entire CC (h) or the anterior, middle, and posterior CC parts (i). (j) The cumulative frequency distribution graph illustrating the relative frequency of myelin internodal lengths in young and aged mice. (k) The histogram representation of data from (h) demonstrating the frequency of myelin sheath lengths in 25 μm bins. *N* (young, aged) = (15 cells; five mice, 17 cells; nine mice, c, e, f), (anterior: five cells, five cells; middle: five cells, seven cells; posterior: five cells, five cells, d, g) (1211 sheaths, 1001 sheaths, h, j, k), (anterior: 411 sheaths, 254 sheaths; middle: 417 sheaths, 412 sheaths; posterior: 383 sheaths, 335 sheaths, i). Data are presented as mean ± SEM (c, d, f, g), median (IQR) (h, i). Student's *t*‐test (c, d, f, g), Pearson's correlation coefficient (e), Mann–Whitney *U*‐test (h, i), and Kolmogorov–Smirnov test (j). *p < 0.05, ***p < 0.001, ****p < 0.0001.

Myelinating mature OLs newly generated in the optic nerve during 120–185 postnatal days have been morphologically different from those newly generated during 30–60 postnatal days and had more and shorter internodes (Young et al. 2013). The myelin internodes were reconstructed and counted with the SNT reconstruction plugin (Fiji software) in young (Figure 4a) and aged (Figure 4b) mice to further investigate if these morphological changes were more pronounced in the CC of aged mice. In contrast to the newly generated myelinating mature OLs in the optic nerve (Young et al. 2013), the number of myelin sheaths per individual newly generated myelinating mature OLs was significantly lower in aged mice (59.7 ± 3.2 sheaths) than in young mice (81.1 ± 3.0 sheaths, *p* < 0.0001) (Figure 4c). The difference in the number of myelin sheaths was significantly lower in aged mice than in young mice in the anterior and middle areas (anterior: 51.6 ± 4.3 vs. 82.2 ± 3.7 sheaths, *p* = 0.0006; middle: 59.4 ± 4.3 vs. 83.4 ± 6.7 sheaths, *p* = 0.0102, respectively) (Figure 4d). In contrast, no significant difference was observed in the number of myelin sheaths between the young and aged mice in the posterior CC area (68.0 ± 6.6 vs. 77.6 ± 5.7 sheaths) (Figure 4d). We evaluated the correlation between the height and the number of sheaths for individual OLs in both young and aged mice to determine the association between the height and the number of sheaths in aged mice. The results revealed that young mice demonstrated a strong positive correlation between the height and the number of sheaths. The height tended to increase (Pearson correlation *R* ^2^ = 0.3210, *p* = 0.0277) as the number of sheaths in young mice elevated (Figure 4e). In contrast, the aged mice appeared to have a highly scattered distribution of the number of sheaths, and no correlation was observed between the height and the number of sheaths (Pearson correlation *R* ^2^ = 0.0002, *p* = 0.9558) (Figure 4e). These results indicate that the decline in aging‐associated myelination may be reflected in the reduced number of myelin sheaths per OL, as well as the reduction being spatially variable within the CC.

We then investigated the internodal length of each OL in aged mice because the nerve conduction velocity is affected by the internodal length (Brill et al. 1977) and decreases with age (Shelly et al. 2023). The average myelin sheath length of the newly generated myelinating mature OLs is significantly shorter in aged mice (49.2 ± 1.7 μm) than in young mice (75.9 ± 2.2 μm, *p* < 0.0001) (Figure 4f). The difference in myelin sheath length per OL in young and aged mice appears consistent throughout the anterior, middle, and posterior CC areas (Figure 4g). Additionally, when the lengths of individual myelin sheaths from all OLs were combined and analyzed as a single dataset, the average myelin sheath length of newly generated myelinating mature OLs was significantly shorter in aged mice (46.1 (36.4–59.0) μm) compared with young mice (72.3 (60.2–89.0) μm, *p* < 0.0001) (Figure 4h). Further, the difference in the individual myelin sheath length per OL in young and aged mice appears consistent throughout the anterior, middle, and posterior CC areas (Figure 4i). The cumulative frequency distribution graph illustrates the relative frequency of the myelin internode lengths in young and aged mice (Kolmogorov–Smirnov test: *D* = 0.5258, *p* < 0.0001). The curve is shifted to the left in the aged mice group compared with the young mice group. This indicates that a higher proportion of internodes are shorter in aged mice than in young mice (Figure 4j). The histogram demonstrates that the highest frequency of myelin sheath lengths was 25–50 μm in aged mice (50.9%), whereas the highest frequency in young mice (44.1%) was 50–75 μm (Figure 4k). In general, internodal length is positively correlated with the diameters of ensheathed axons (Hess and Young 1949). To investigate this relationship, we measured fiber diameters of myelin sheaths formed by the newly generated myelinating OLs in the CC of young and aged mice because fiber diameter reflects axon diameter and myelin thickness, and larger axons tend to have thicker myelin sheaths. We found no significant difference in the average fiber diameter of newly generated myelinating mature OLs between aged and young mice (Figure S5). These results indicate that a greater height, a fewer number of myelin sheaths, and shorter internode lengths characterized the morphological changes of newly generated myelinating mature OLs during aging.

## 4 Discussion

In this study, we utilized *Pdgfra‐CreER* ^*T2*^:*Tau‐mGFP* double transgenic mice to selectively label the newly generated OLs following tamoxifen injection of aged mice, focusing on CC. Our research indicated that aging significantly affected the accumulation of newly generated OLs that differentiated into myelinating mature OLs, with their number being significantly lower in aged mice than in young mice throughout the CC. Moreover, newly generated myelinating mature OLs in aged mice demonstrated significantly greater height, significantly shorter myelin internodes, and a significantly fewer number of myelin sheaths compared with those in young mice. Our results indicate that the lower number and distinct morphological alterations observed in newly generated OLs in aged mice may contribute to the disruptions in myelin maintenance and myelination related to aging.

Consistent with a previous study (Young et al. 2013), our results indicate that this transgenic mouse model effectively labeled newly generated OLs in young and aged mice following tamoxifen‐induced Cre recombination. GFP expression in the CC was found specifically in tamoxifen‐treated mice. This labeling, driven by the *Tau* promoter, is restricted to OLs that are derived from *Pdgfra*‐positive OPCs, thereby enabling precise tracking of OL generation, survival, and maturation. Immunostaining further validated the lineage specificity because GFP colocalized with OL markers (Olig2 and CC1) but not with astrocyte (GFAP) or microglia (Iba1) markers. It has been suggested that the recombination efficiency between young and aged mice is not significantly different following tamoxifen administration (Kellogg et al. 2023). This reliable and specific labeling system of newly generated OLs enables detailed analysis of age‐related alterations in the accumulation rate and the morphological characteristics of newly generated mature OLs.

We revealed that the percentage of newly generated OLs that differentiate into myelinating mature OLs was significantly lower in aged mice, with this decline consistent across all CC areas. OPCs play a crucial role in maintaining myelination in the adult brain (Psachoulia et al. 2009; Vanzulli et al. 2020), but the processes of their division and the differentiation of newly generated OLs with aging remain incompletely understood. Our results indicated that approximately 54% of the newly generated OLs in the young (8 + 2 weeks) group differentiated into premyelinating and myelinating mature OLs, whereas this proportion decreased in the aged group (78 + 2 weeks, 6%; 78 + 4 weeks, 12%). These results support the concept that the OPC differentiation rate declines with age (Dimovasili et al. 2023). Additionally, extending the recombination period in aged mice did not significantly improve the accumulation of the newly generated myelinating mature OLs, indicating an intrinsic limitation in the differentiation capacity of aged OPCs (de la Fuente et al. 2020). The molecular mechanisms that disrupt the OPC's function in aging, including cellular senescence, telomere shortening, epigenetic regulation, and mitochondrial dysfunction with oxidative stress, have been reported (Jaskelioff et al. 2011; Leenders et al. 2024; Neumann et al. 2019). OPCs have significant metabolic demands during differentiation (Rosko et al. 2019). However, oxidative phosphorylation and respiratory electron transport pathways are downregulated in the aged OPCs (Luan et al. 2021). These changes are characteristic of aging and may contribute to limited differentiation capacity. A previous study demonstrated that the proliferation rate of OPCs decreases with age, as shown by a substantial increase in the cell cycle time (T_C_) for OPCs in the CC, elevating from 2 days at P6 to 3 days at P21, 10 days at P60, and reaching 70 days at P240 and P540 (Psachoulia et al. 2009; Young et al. 2013). These reports indicate that aging is associated with impaired differentiation and reduced proliferation of OPCs, which are crucial to maintaining myelination in the adult brain, and OPC impairment in aging causes insufficient remyelination (Leenders et al. 2024; Woodruff et al. 2004).

Our results indicated that the morphology of newly generated myelinating mature OLs and their myelin sheaths was altered in aged mice. These OLs in aged mice demonstrate greater height compared with young mice. OL height is greater in aged mice and remains consistently greater across all CC areas. A maximum of 70% of axons remains unmyelinated by 8 months of age in the mouse CC, providing abundant nearby unmyelinated axons (Sturrock 1980; Tripathi et al. 2017). Thus, young OLs do not need to extend processes because several unmyelinated axons are within proximity to their cell bodies. The newly generated OLs in the CC produced additional myelin sheaths for previously unmyelinated or partially myelinated axons, rather than replacing the dying OLs, during the first 8 months of life (Tripathi et al. 2017). The increased height of newly generated OLs in aged mice may indicate a necessity to extend processes over longer distances to reach unmyelinated axons for myelination, as the surrounding axons remain myelinated. We propose this enlargement to be one of the hallmark morphological changes observed in newly generated myelinating OLs during aging.

Additionally, newly generated mature myelinating OLs in aged mice demonstrated a reduced number of myelin sheaths and shorter internodal lengths compared with those in young mice. This is consistent with the result that the production of OLs with impaired morphology in aging causes total myelin loss in the advanced stages of aging (Chapman and Hill 2020). Producing new OLs and forming new myelin to maintain the original myelin pattern may be necessary to replace myelin that is lost with age. Additionally, we revealed that the number of myelin sheaths formed by newly generated OLs in the posterior region of aged mice was comparable with that in younger mice. The CC has distinct regions for different axon types: those connecting the prefrontal cortex pass through the anterior part, whereas axons connecting the motor and sensory regions cross through the middle region, and axons from the parietal and occipital regions run through the posterior part (Fabri and Polonara 2023). Regional factors or variations in axon types in each area may affect the changes in the number of myelin sheaths in aged mice; the posterior part of the CC in aged mice may lose myelin more frequently and/or have more unmyelinated axons, which will promote myelin formation. This is particularly crucial because myelin degeneration in the CC occurs throughout the lifespan and becomes more pronounced during normal aging (Parandavar et al. 2024).

A decrease in the number of sheaths alongside increased OL height in aged mice may be influenced by differences in exposed axon availability and changes in the intrinsic environment. Age‐related alterations in metabolic support, including mitochondrial function (Bame and Hill 2024; Neumann et al. 2019), may impact OL morphogenesis. The metabolic support for myelin production may be insufficient for OLs in aged mice to extend longer processes, potentially limiting their ability to generate multiple processes capable of forming myelin at their tips, ultimately leading to a reduced number of myelin sheaths.

We revealed that the internode length of newly formed myelin sheaths was significantly shorter in aged mice than in young mice across all CC areas. The internode length is one of the factors that control the conduction speed (Swire et al. 2021; Young et al. 2013), and the length of new myelin internodes is shorter during myelination than that of existing ones (Gledhill and McDonald 1977). This indicates the involvement of the length of newly formed myelin sheaths in the functional regulation of neuronal circuits in aged mice, as myelin maintenance depends on OL replacement (Chapman and Hill 2020). Internodal length is positively correlated with axon diameter (Hess and Young 1949); therefore, smaller diameter axons available for myelination in the aged CC may contribute to the observed differences in internodal lengths. However, our light microscopy analysis revealed no significant difference in the diameter of myelin sheaths formed by newly generated myelinating mature OLs in aged and young mice. The dysfunction in the synthesis of myelin components (de la Fuente et al. 2020) may cause shorter myelin internodes as well as a reduced number of myelin sheaths of newly generated OLs in aged mice. In addition to the possibility of different fiber diameters, our results indicate a reduced capacity for efficient axonal insulation, which could cause slower signal conduction in the aging brain, potentially contributing to cognitive decline (Ibanez et al. 2024).

A limitation of this study is the difficulty of obtaining a sufficient sample size of individual OLs in aged mice due to the reduced number of newly generated myelinating OLs and the difficulty in determining the whole cells within the ROI at 100 μm‐thickness tissue sections. The challenge originates from the clustering of newly generated OLs in young mice, making individual cell identification difficult—a consequence of the higher rate of accumulation observed in these mice. Another limitation is that our study focused on newly generated OLs only in the CC, leaving the question of regional variation in aging uninvestigated. Further research is warranted to examine the differences in terms of the differentiation, maturation, and morphology of these new OLs across different brain regions. The third limitation is the use of 18‐month‐old mice, which may raise questions about the changes occurring in advanced aging. We revealed that using 18‐month‐old mice is suitable for studying the alterations in individual OLs in aged mice because we needed to be cautious about the survival rate under advanced aging, especially following tamoxifen injections. Proteome analysis in aged OPCs (15–18 months) revealed that cholesterol biosynthesis, transcription factors, and cell cycle proteins decreased (de la Fuente et al. 2020). Additionally, this age point enables us to observe significant differences in accumulation rates and morphological changes in mature OLs, providing information without complications.

In conclusion, our research provides compelling evidence that aging significantly impairs the accumulation of newly generated OLs in the CC and causes morphological changes in these cells. These results indicate the complex interplay between OL differentiation, morphology, and myelination in the aging brain and highlight the importance of myelination in the aged brain. The reduced number and altered functionality of myelinating OLs may contribute to the delayed myelination and impaired neuronal communication observed in the aging brain, potentially causing disruptions in the neural networks that are crucial for memory, learning, and other cognitive processes (Bowley et al. 2010). Future studies are warranted to further elucidate the molecular mechanisms driving these age‐related changes and investigate therapeutic strategies to improve myelination and enhance cognitive outcomes in aging populations. Furthermore, although it would require a new approach, studying the morphology of newly generated OLs following demyelination in young and aged brains would be valuable to better understand the factors regulating OL morphology, such as environmental influences including the abundance of axons available for myelination.

## Author Contributions

S.L., R.Y., Y.O., and N.O. conceived and designed the experiments and acquired funding. S.L. performed the experiments and analyzed the data. S.L., R.Y., and N.O. wrote the original draft. S.L., R.Y., Y.O., and N.O. reviewed and edited the manuscript.

## Ethics Statement

This study has been approved by the Institutional Animal Care and Use Committee and the Committee for Genetic Recombination Experiments of Jichi Medical University (Approval No. 23005‐05).

## Conflicts of Interest

The authors declare no conflicts of interest.

## Supporting information


**Data S1.** Supporting information.

## Data Availability

The data that support the findings of this study are available from the corresponding author upon reasonable request.

## References

[glia70070-bib-0001] Arshadi, C. , U. Gunther , M. Eddison , K. I. S. Harrington , and T. A. Ferreira . 2021. “SNT: A Unifying Toolbox for Quantification of Neuronal Anatomy.” Nature Methods 18, no. 4: 374–377. 10.1038/s41592-021-01105-7. 33795878

[glia70070-bib-0002] Bame, X. , and R. A. Hill . 2024. “Mitochondrial Network Reorganization and Transient Expansion During Oligodendrocyte Generation.” Nature Communications 15, no. 1: 6979. 10.1038/s41467-024-51016-2. PMC11324877 39143079

[glia70070-bib-0003] Bercury, K. K. , and W. B. Macklin . 2015. “Dynamics and Mechanisms of CNS Myelination.” Developmental Cell 32, no. 4: 447–458. 10.1016/j.devcel.2015.01.016. 25710531 PMC6715306

[glia70070-bib-0004] Bowley, M. P. , H. Cabral , D. L. Rosene , and A. Peters . 2010. “Age Changes in Myelinated Nerve Fibers of the Cingulate Bundle and Corpus Callosum in the Rhesus Monkey.” Journal of Comparative Neurology 518, no. 15: 3046–3064. 10.1002/cne.22379. 20533359 PMC2889619

[glia70070-bib-0005] Boyle, M. E. T. , E. O. Berglund , K. K. Murai , L. Weber , E. Peles , and B. Ranscht . 2001. “Contactin Orchestrates Assembly of the Septate‐Like Junctions at the Paranode in Myelinated Peripheral Nerve.” Neuron 30, no. 2: 385–397. 10.1016/S0896-6273(01)00296-3. 11395001

[glia70070-bib-0006] Brill, M. H. , S. G. Waxman , J. W. Moore , and R. W. Joyner . 1977. “Conduction Velocity and Spike Configuration in Myelinated Fibres: Computed Dependence on Internode Distance.” Journal of Neurology, Neurosurgery, and Psychiatry 40, no. 8: 769–774. 10.1136/jnnp.40.8.769. 925697 PMC492833

[glia70070-bib-0007] Cantuti‐Castelvetri, L. , D. Fitzner , M. Bosch‐Queralt , et al. 2018. “Defective Cholesterol Clearance Limits Remyelination in the Aged Central Nervous System.” Science 359, no. 6376: 684–688. 10.1126/science.aan4183. 29301957

[glia70070-bib-0008] Chapman, T. W. , and R. A. Hill . 2020. “Myelin Plasticity in Adulthood and Aging.” Neuroscience Letters 715: 134645. 10.1016/j.neulet.2019.134645. 31765728 PMC6981290

[glia70070-bib-0009] de la Fuente, A. G. , R. M. L. Queiroz , T. Ghosh , et al. 2020. “Changes in the Oligodendrocyte Progenitor Cell Proteome With Ageing.” Molecular and Cellular Proteomics 19, no. 8: 1281–1302. 10.1074/mcp.RA120.002102. 32434922 PMC8015006

[glia70070-bib-0010] Dimovasili, C. , A. E. Fair , I. R. Garza , et al. 2023. “Aging Compromises Oligodendrocyte Precursor Cell Maturation and Efficient Remyelination in the Monkey Brain.” Geroscience 45, no. 1: 249–264. 10.1007/s11357-022-00621-4. 35930094 PMC9886778

[glia70070-bib-0011] Fabri, M. , and G. Polonara . 2023. “Functional Topography of the Corpus Callosum as Revealed by fMRI and Behavioural Studies of Control Subjects and Patients With Callosal Resection.” Neuropsychologia 183: 108533. 10.1016/j.neuropsychologia.2023.108533. 36906223

[glia70070-bib-0012] Fields, R. D. 2014. “Neuroscience. Myelin–More Than Insulation.” Science 344, no. 6181: 264–266. 10.1126/science.1253851. 24744365 PMC5017201

[glia70070-bib-0013] Fjell, A. M. , L. T. Westlye , H. Grydeland , et al. 2014. “Accelerating Cortical Thinning: Unique to Dementia or Universal in Aging?” Cerebral Cortex 24, no. 4: 919–934. 10.1093/cercor/bhs379. 23236213 PMC3948495

[glia70070-bib-0014] Ford, M. C. , O. Alexandrova , L. Cossell , et al. 2015. “Tuning of Ranvier Node and Internode Properties in Myelinated Axons to Adjust Action Potential Timing.” Nature Communications 6: 8073. 10.1038/ncomms9073. PMC4560803 26305015

[glia70070-bib-0015] Gledhill, R. F. , and W. I. McDonald . 1977. “Morphological Characteristics of Central Demyelination and Remyelination: A Single‐Fiber Study.” Annals of Neurology 1, no. 6: 552–560. 10.1002/ana.410010607. 883767

[glia70070-bib-0016] Hess, A. , and J. Z. Young . 1949. “Correlation of Internodal Length and Fibre Diameter in the Central Nervous System.” Nature 164, no. 4168: 490–491. 10.1038/164490a0. 18140456

[glia70070-bib-0017] Hill, R. A. , A. M. Li , and J. Grutzendler . 2018. “Lifelong Cortical Myelin Plasticity and Age‐Related Degeneration in the Live Mammalian Brain.” Nature Neuroscience 21, no. 5: 683–695. 10.1038/s41593-018-0120-6. 29556031 PMC5920745

[glia70070-bib-0018] Hippenmeyer, S. , E. Vrieseling , M. Sigrist , et al. 2005. “A Developmental Switch in the Response of DRG Neurons to ETS Transcription Factor Signaling.” PLoS Biology 3, no. 5: e159. 10.1371/journal.pbio.0030159. 15836427 PMC1084331

[glia70070-bib-0019] Ibanez, S. , N. Sengupta , J. I. Luebke , K. Wimmer , and C. M. Weaver . 2024. “Myelin Dystrophy Impairs Signal Transmission and Working Memory in a Multiscale Model of the Aging Prefrontal Cortex.” eLife 12: RP90964. 10.7554/eLife.90964.3. 39028036 PMC11259433

[glia70070-bib-0020] Jaskelioff, M. , F. L. Muller , J. H. Paik , et al. 2011. “Telomerase Reactivation Reverses Tissue Degeneration in Aged Telomerase‐Deficient Mice.” Nature 469, no. 7328: 102–106. 10.1038/nature09603. 21113150 PMC3057569

[glia70070-bib-0021] Kellogg, C. M. , K. Pham , S. Ko , et al. 2023. “Specificity and Efficiency of Tamoxifen‐Mediated Cre Induction Is Equivalent Regardless of Age.” IScience 26, no. 12: 108413. 10.1016/j.isci.2023.108413. 38058312 PMC10696116

[glia70070-bib-0022] Lasiene, J. , A. Matsui , Y. Sawa , F. Wong , and P. J. Horner . 2009. “Age‐Related Myelin Dynamics Revealed by Increased Oligodendrogenesis and Short Internodes.” Aging Cell 8, no. 2: 201–213. 10.1111/j.1474-9726.2009.00462.x. 19338498 PMC2703583

[glia70070-bib-0023] Leenders, F. , L. Koole , H. Slaets , A. Tiane , D. V. D. Hove , and T. Vanmierlo . 2024. “Navigating Oligodendrocyte Precursor Cell Aging in Brain Health.” Mechanisms of Ageing and Development 220: 111959. 10.1016/j.mad.2024.111959. 38950628

[glia70070-bib-0024] Luan, W. , X. Qi , F. Liang , et al. 2021. “Microglia Impede Oligodendrocyte Generation in Aged Brain.” Journal of Inflammation Research 14: 6813–6831. 10.2147/jir.S338242. 34924766 PMC8674668

[glia70070-bib-0025] Mathews, E. S. , and B. Appel . 2016. “Chapter 3—Oligodendrocyte Differentiation.” In Methods in Cell Biology, edited by H. W. Detrich , M. Westerfield , and L. I. Zon , vol. 134, 69–96. Academic Press. 10.1016/bs.mcb.2015.12.004 27312491

[glia70070-bib-0026] Neumann, B. , R. Baror , C. Zhao , et al. 2019. “Metformin Restores CNS Remyelination Capacity by Rejuvenating Aged Stem Cells.” Cell Stem Cell 25, no. 4: 473–485.e478. 10.1016/j.stem.2019.08.015. 31585093 PMC6863391

[glia70070-bib-0027] Osanai, Y. , B. Battulga , R. Yamazaki , et al. 2022. “Dark Rearing in the Visual Critical Period Causes Structural Changes in Myelinated Axons in the Adult Mouse Visual Pathway.” Neurochemical Research 47, no. 9: 2815–2825. 10.1007/s11064-022-03689-8. 35933550

[glia70070-bib-0028] Osso, L. A. , and E. G. Hughes . 2024. “Dynamics of Mature Myelin.” Nature Neuroscience 27, no. 8: 1449–1461. 10.1038/s41593-024-01642-2. 38773349 PMC11515933

[glia70070-bib-0029] Parandavar, E. , M. Shafizadeh , S. Ahmadian , and M. Javan . 2024. “Long‐Term Demyelination and Aging‐Associated Changes in Mice Corpus Callosum; Evidence for the Role of Accelerated Aging in Remyelination Failure in a Mouse Model of Multiple Sclerosis.” Aging Cell 23, no. 9: e14211. 10.1111/acel.14211. 38804500 PMC11488340

[glia70070-bib-0030] Pitman, K. A. , R. Ricci , R. Gasperini , et al. 2020. “The Voltage‐Gated Calcium Channel CaV1.2 Promotes Adult Oligodendrocyte Progenitor Cell Survival in the Mouse Corpus Callosum but Not Motor Cortex.” Glia 68, no. 2: 376–392. 10.1002/glia.23723. 31605513 PMC6916379

[glia70070-bib-0031] Psachoulia, K. , F. Jamen , K. M. Young , and W. D. Richardson . 2009. “Cell Cycle Dynamics of NG2 Cells in the Postnatal and Ageing Brain.” Neuron Glia Biology 5, no. 3–4: 57–67. 10.1017/S1740925X09990354. 20346197 PMC6329448

[glia70070-bib-0032] R Core Team . 2025. R: A Language and Environment for Statistical Computing. R Foundation for Statistical Computing. https://www.R‐project.org/.

[glia70070-bib-0033] Rios, J. C. , C. V. Melendez‐Vasquez , S. Einheber , et al. 2000. “Contactin‐Associated Protein (Caspr) and Contactin Form a Complex That Is Targeted to the Paranodal Junctions During Myelination.” Journal of Neuroscience 20, no. 22: 8354–8364. 10.1523/jneurosci.20-22-08354.2000. 11069942 PMC6773165

[glia70070-bib-0034] Rivers, L. E. , K. M. Young , M. Rizzi , et al. 2008. “PDGFRA/NG2 Glia Generate Myelinating Oligodendrocytes and Piriform Projection Neurons in Adult Mice.” Nature Neuroscience 11, no. 12: 1392–1401. 10.1038/nn.2220. 18849983 PMC3842596

[glia70070-bib-0035] Roland, J. L. , A. Z. Snyder , C. D. Hacker , et al. 2017. “On the Role of the Corpus Callosum in Interhemispheric Functional Connectivity in Humans.” Proceedings of the National Academy of Sciences of the United States of America 114, no. 50: 13278–13283. 10.1073/pnas.1707050114. 29183973 PMC5740665

[glia70070-bib-0036] Rosko, L. , V. N. Smith , R. Yamazaki , and J. K. Huang . 2019. “Oligodendrocyte Bioenergetics in Health and Disease.” Neuroscientist 25, no. 4: 334–343. 10.1177/1073858418793077. 30122106 PMC6745601

[glia70070-bib-0037] Sampaio‐Baptista, C. , and H. Johansen‐Berg . 2017. “White Matter Plasticity in the Adult Brain.” Neuron 96, no. 6: 1239–1251. 10.1016/j.neuron.2017.11.026. 29268094 PMC5766826

[glia70070-bib-0038] Sams, E. C. 2021. “Oligodendrocytes in the Aging Brain.” Neuronal Signaling 5, no. 3: NS20210008. 10.1042/NS20210008. 34290887 PMC8264650

[glia70070-bib-0039] Shelly, S. , R. Ramon‐Gonen , P. Paul , et al. 2023. “Nerve Conduction Differences in a Large Clinical Population: The Role of Age and Sex.” Journal of Neuromuscular Diseases 10, no. 5: 925–935. 10.3233/JND-230052. 37545257 PMC10578272

[glia70070-bib-0040] Sim, F. J. , C. Zhao , J. Penderis , and R. J. Franklin . 2002. “The Age‐Related Decrease in CNS Remyelination Efficiency Is Attributable to an Impairment of Both Oligodendrocyte Progenitor Recruitment and Differentiation.” Journal of Neuroscience 22, no. 7: 2451–2459. 10.1523/JNEUROSCI.22-07-02451.2002. 11923409 PMC6758320

[glia70070-bib-0041] Sturrock, R. R. 1980. “Myelination of the Mouse Corpus Callosum.” Neuropathology and Applied Neurobiology 6, no. 6: 415–420. 10.1111/j.1365-2990.1980.tb00219.x. 7453945

[glia70070-bib-0042] Swire, M. , P. Assinck , P. A. McNaughton , D. A. Lyons , C. Ffrench‐Constant , and M. R. Livesey . 2021. “Oligodendrocyte HCN2 Channels Regulate Myelin Sheath Length.” Journal of Neuroscience 41, no. 38: 7954–7964. 10.1523/JNEUROSCI.2463-20.2021. 34341156 PMC8460148

[glia70070-bib-0043] Tanaka, T. , N. Ohno , Y. Osanai , et al. 2021. “Large‐Scale Electron Microscopic Volume Imaging of Interfascicular Oligodendrocytes in the Mouse Corpus Callosum.” Glia 69, no. 10: 2488–2502. 10.1002/glia.24055. 34165804

[glia70070-bib-0044] Tripathi, R. B. , M. Jackiewicz , I. A. McKenzie , E. Kougioumtzidou , M. Grist , and W. D. Richardson . 2017. “Remarkable Stability of Myelinating Oligodendrocytes in Mice.” Cell Reports 21, no. 2: 316–323. 10.1016/j.celrep.2017.09.050. 29020619 PMC5643547

[glia70070-bib-0045] Ulloa‐Navas, M. J. , P. Perez‐Borreda , R. Morales‐Gallel , et al. 2021. “Ultrastructural Characterization of Human Oligodendrocytes and Their Progenitor Cells by Pre‐Embedding Immunogold.” Frontiers in Neuroanatomy 15: 696376. 10.3389/fnana.2021.696376. 34248510 PMC8262677

[glia70070-bib-0046] Vanzulli, I. , M. Papanikolaou , I. C. De‐La‐Rocha , et al. 2020. “Disruption of Oligodendrocyte Progenitor Cells Is an Early Sign of Pathology in the Triple Transgenic Mouse Model of Alzheimer's Disease.” Neurobiology of Aging 94: 130–139. 10.1016/j.neurobiolaging.2020.05.016. 32619874 PMC7453384

[glia70070-bib-0047] Woodruff, R. H. , M. Fruttiger , W. D. Richardson , and R. J. Franklin . 2004. “Platelet‐Derived Growth Factor Regulates Oligodendrocyte Progenitor Numbers in Adult CNS and Their Response Following CNS Demyelination.” Molecular and Cellular Neuroscience 25, no. 2: 252–262. 10.1016/j.mcn.2003.10.014. 15019942

[glia70070-bib-0048] Young, K. M. , K. Psachoulia , R. B. Tripathi , et al. 2013. “Oligodendrocyte Dynamics in the Healthy Adult CNS: Evidence for Myelin Remodeling.” Neuron 77, no. 5: 873–885. 10.1016/j.neuron.2013.01.006. 23473318 PMC3842597

