# Supplementary material for: Reduced Accumulation Rate and Morphological Changes of Newly Generated Myelinating Oligodendrocytes in the Corpus Callosum of Aged Mice
Source: Glia. 2025 Aug 1;73(11):2322–34. doi: 10.1002/glia.70070 (PMC12436990; doi:10.1002/glia.70070)
Supplement: Supplementary file 1 — Data S1. Supporting information. [file GLIA-73-2322-s001.pdf]

Supporting Information

Reduced Accumulation Rate and Morphological Changes of Newly Generated Myelinating Oligodendrocytes in the Corpus Callosum of Aged Mice

Data S1

Methods S1

Confirmation of specific labeling of newly generated oligodendrocytes (OLs)

Brain slices were immunostained for GFP, GFAP, and Olig2 to confirm that no GFP+ cells colocalized with astrocytes. Additionally, immunostaining for GFP, Iba1, and CC1 was performed to confirm that no GFP+ cells colocalized with microglia.

For astrocyte confirmation, all GFP+ cells in the corpus callosum were examined using a z-stack series, and GFP+GFAP− and GFP+GFAP+ cells were counted in different regions of the corpus callosum (N = one mouse per group: young (8-week-old) and aged (78-week-old), with three brain slices analyzed per mouse).

For microglia confirmation, all GFP+ cells in the corpus callosum were also examined using a z-stack series, and GFP+Iba1− and GFP+Iba1+ cells were counted in different regions of the corpus callosum (N = one mouse per group: young (8-week-old) and aged (78-week-old), with three brain slices analyzed per mouse).

Analysis of the proportion of GFP+ cells that express the OPC marker NG2

Brain slices were immunostained for GFP, NG2, and CC1 to confirm the recombined OPCs (GFP+NG2+cells). For each 60-image z-stack series of 30 µm (0.5 µm spacing), images were obtained from a 2000-µm vertical region lateral to the brain midline of the anterior CC.

From this set, 10 images were analyzed. NG2+GFP+ cells, NG2+ cells, and GFP+ cells within a 100 μm-thick coronal section were counted using Fiji.

Fiber diameter measurement

Fiber diameter measurement was performed as described previously with minor modifications (Tanaka et al. 2021). The z-stack images of each myelin sheath of individual OLs were compressed into single-plane images unless they overlapped with another myelin sheath. In cases where myelin sheaths overlapped, the fiber diameter was measured individually from the z- stack series images. Fiber diameter was measured at six points along the length of each myelin internode by dividing it into six equal parts using the formula: Distance for measurement position (μm) = (myelin internode length (μm) −2) / 5. The first and last points were positioned at 1 μm from the rims of the myelin sheath. The second to fifth points were evenly spaced along the internode, with the distance between them determined by the value obtained from the formula. The average fiber diameter was then calculated based on these measurements.

For each individual OL, five myelin internodes were measured, including the outermost myelin sheath on both the dorsal and ventral sides, as well as one internode in the middle, one between the middle and the outermost dorsal sheath, and one between the middle and the outermost ventral sheath (Figure S5).

Reference

Tanaka, T., N. Ohno, Y. Osanai, et al. 2021. “Large-Scale Electron Microscopic Volume Imaging of Interfascicular Oligodendrocytes in the Mouse Corpus Callosum.” *Glia* 69, no. 10: 2488–2502. http://doi:10.1002/glia.24055.

Table S1. Quantitative data showing that none of the GFP+ cells colocalized with Iba1 or GFAP.

Cells		Entire CC	Anterior CC	Middle CC	Posterior CC
GFP+ Iba1+	Young	0	0	0	0
	Aged	0	0	0	0
GFP+ Iba1−	Young	66	23	24	19
	Aged	12	4	3	5
GFP+ GFAP+	Young	0	0	0	0
	Aged	0	0	0	0
GFP+ GFAP−	Young	63	21	22	20
	Aged	13	6	4	3

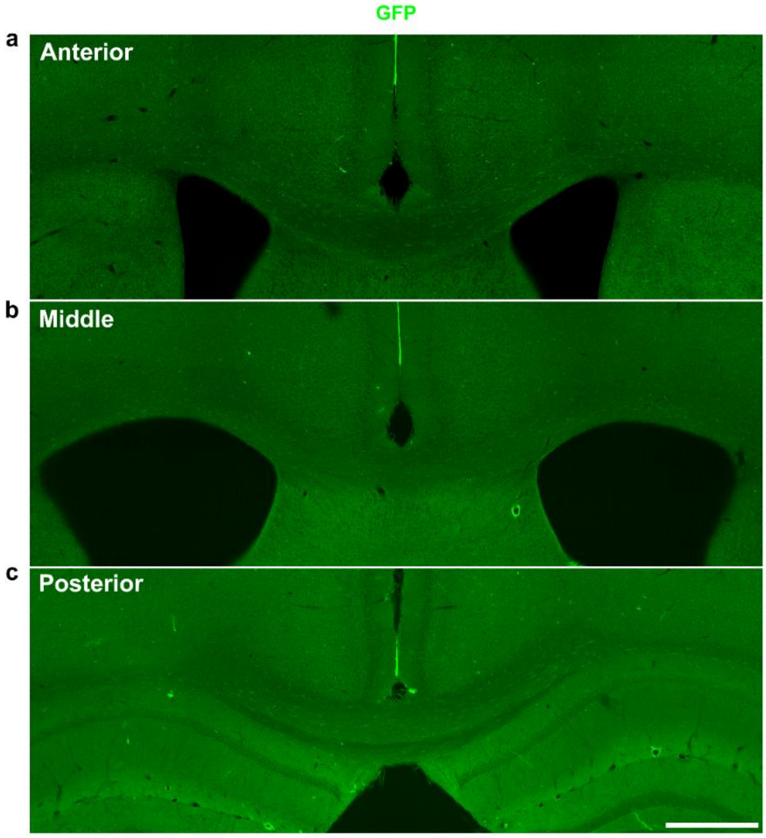

Figure S1. Confirmation of no expression of GFP in *Pdgfra-CreER^{T2}:Tau-mGFP* double transgenic mice without tamoxifen injection. (a-c) The representative fluorescent images of slices from the young transgenic mice at low magnifications, confirming no GFP-positive cells without tamoxifen injection. The slices were obtained from 3 different levels in the rostral-caudal axis, anterior (bregma 0.37 to 0.73) (a), middle (bregma -0.47 to -0.83) (b) and posterior (bregma -1.67 to -2.03) (c). CC: corpus callosum, Cpu: Caudate putamen, Ctx: cortex, HP: hippocampus, LV: lateral ventricle, vhc: ventral hippocampal commissure. Scale bars: 500 µm.

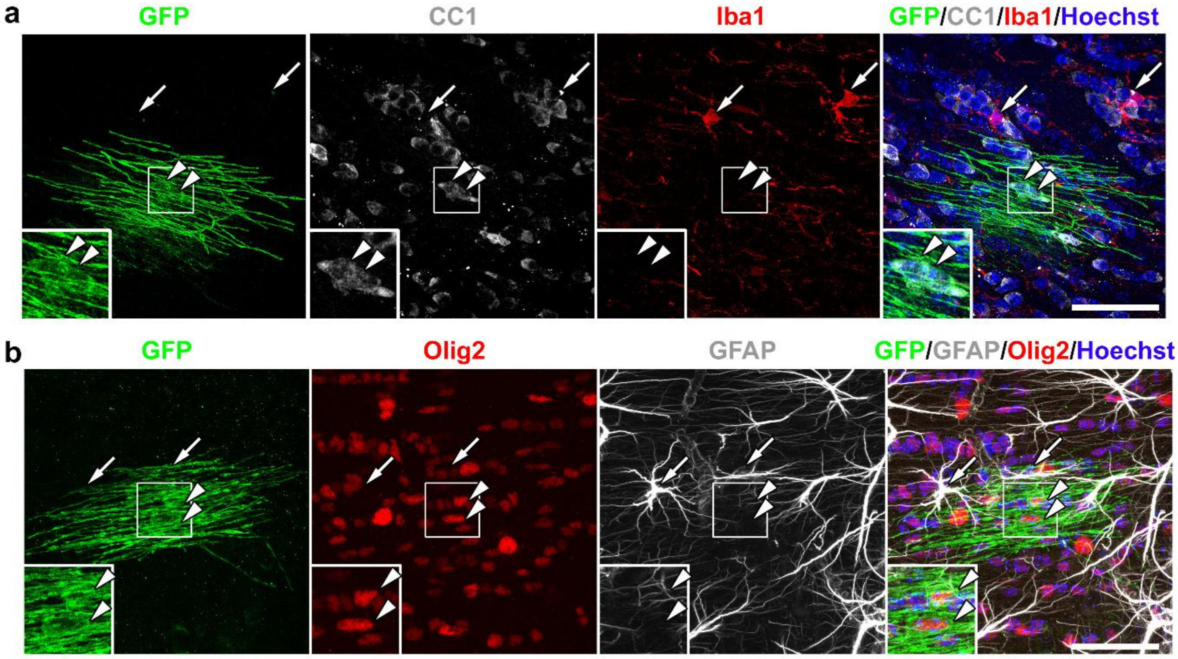

Figure S2. Confirmation of specific labeling of newly generated oligodendrocytes. (a, b) Immunostaining for GFP (a, b, green) along with CC1 (mature OL marker, a, white), Olig2 (OL lineage marker, b, red), Iba1 (microglia marker, a, red), and GFAP (astrocyte marker, b, white) from young transgenic mice showed that GFP immunoreactivity colocalized with a fraction of Olig2-positive (b, arrowheads) and CC1-positive (a, arrowheads) OLs, but not with GFAP-positive astrocytes (a, arrows) or Iba1-positive microglia (b, arrows). Scale bars: 50 µm.

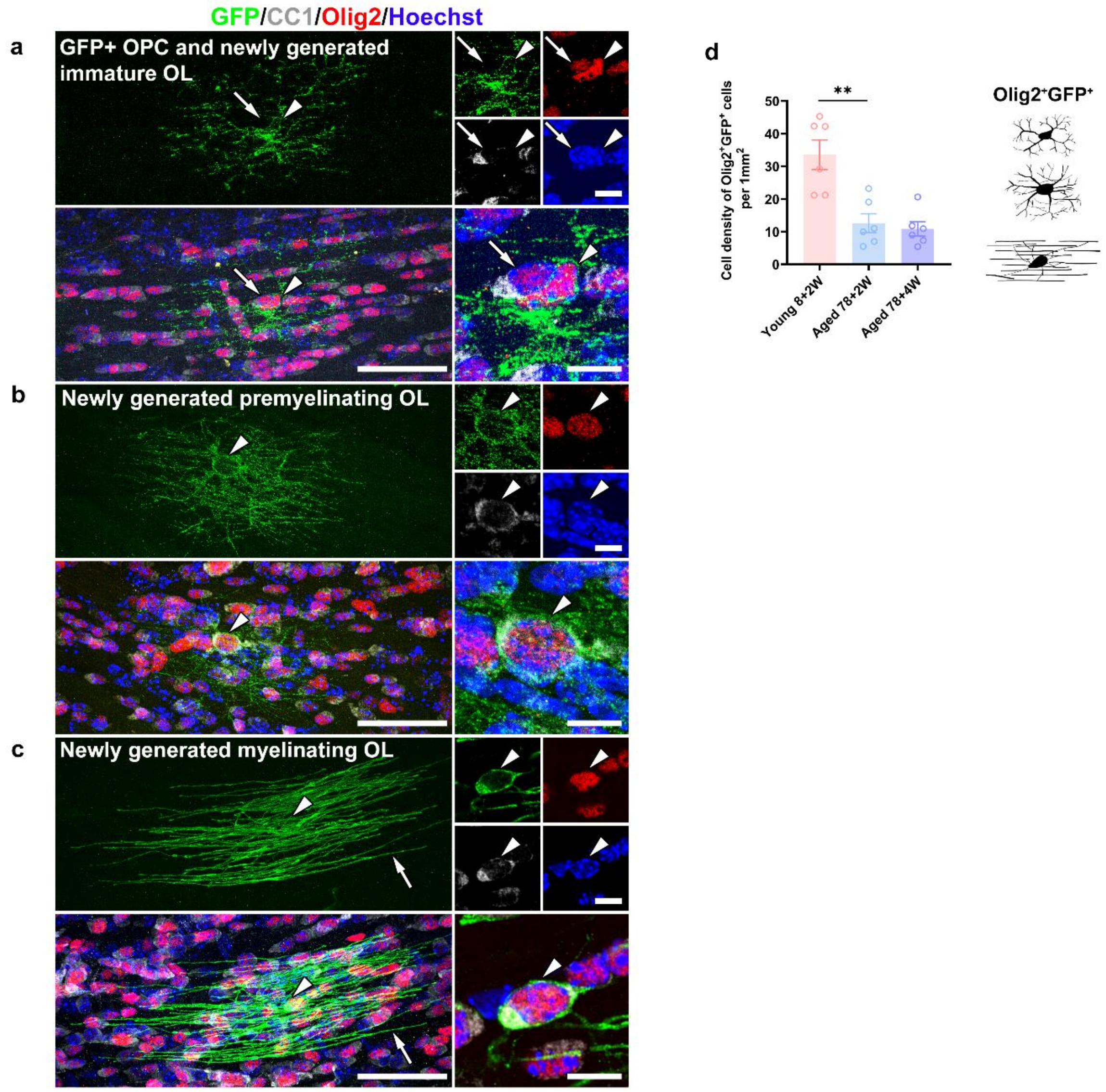

Figure S3. Characteristics of newly generated OLs in the corpus callosum of young *Pdgfra-CreER^{T2}:Tau-mGFP* double transgenic mice. (a-c) Immunostaining for GFP (green), Olig2 (red) and CC1 (white) in the corpus callosum (CC) of young transgenic mice. One of the GFP+ oligodendrocyte progenitor cells (OPCs) and a newly generated immature OL are positive for GFP and Olig2 but negative for CC1 (a, arrowheads) and are located beside a GFP-negative mature OL, which is positive for Olig2 and CC1 (a, arrows). The newly generated premyelinating mature OL (b, arrowheads) and newly generated myelinating mature OL (c, arrowheads) are immunopositive for GFP, Olig2 and CC1, and the latter has straight GFP-

positive processes corresponding to myelin internodes (c, arrows). (d) The density of cells positive for GFP and Olig2 in the corpus callosum of the three groups, 8 + 2 weeks, 78 + 2 weeks, and 78 + 4 weeks. N = six mice per group; three males and three females; three coronal slices from each mouse were combined. Nuclei are counterstained with Hoechst (blue). Scale bars: 50 (left) and 10 (right) µm. Data are expressed as the mean ± SEM (d). One-way ANOVA (d). $^{**}p < 0.01$.

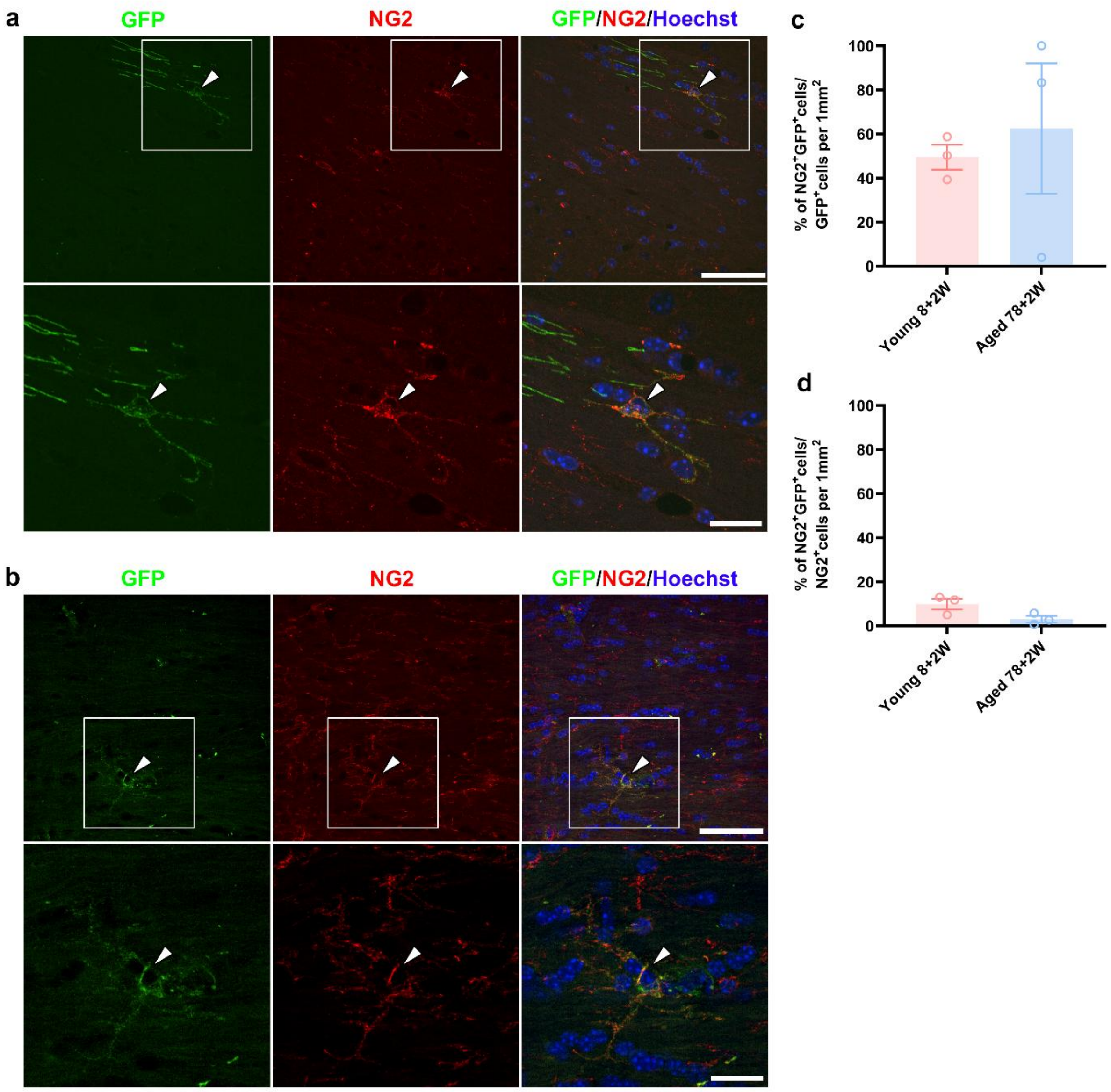

Figure S4. Confirmation of recombined OPCs, including GFP+ OPCs and newly generated immature OLs. (a, b) Immunostaining for GFP and NG2 (OPC marker) in young (a) and aged (b) *Pdgfra-CreER^{T2}:Tau-mGFP* double transgenic mice showed that GFP immunoreactivity colocalized with a fraction of NG2-positive cells (a, b, arrowheads). (c) The proportion of recombined OPCs (NG2+GFP+) amongst GFP+ cells in the anterior of the corpus callosum. (d) The proportion of recombined OPCs (NG2+GFP+) amongst NG2+ cells in the anterior of the corpus callosum. N = three mice per group; one coronal slice per mouse. Data are expressed as the mean ± SEM (c, d). Scale bars: 50 µm (upper) and 20 µm (lower) (a, b).

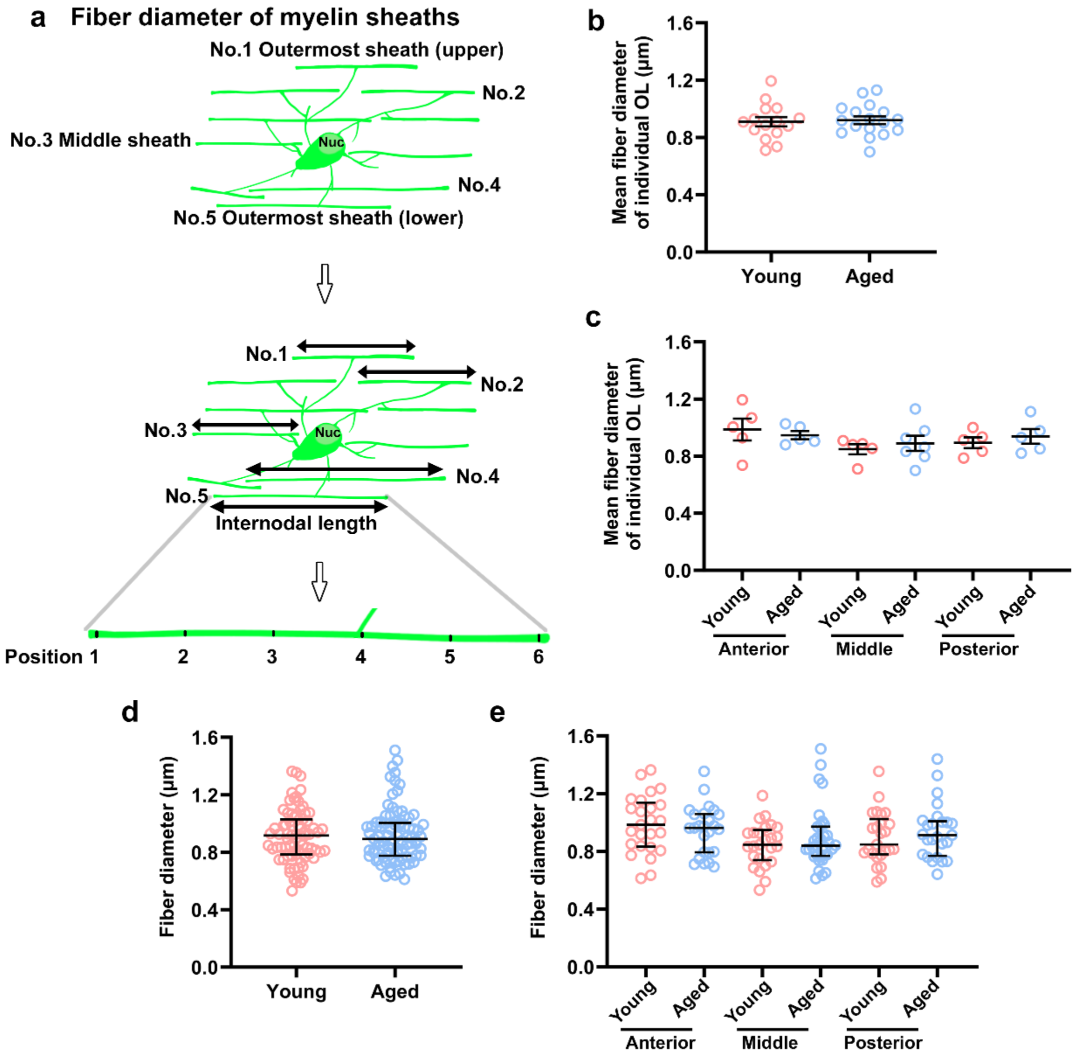

Figure S5. Fiber diameter analysis of individual myelin sheaths of newly generated myelinating mature OLs in the corpus callosum of *Pdgfra-CreER^{T2}:Tau-mGFP* double transgenic mice. (a) Schemes illustrating myelinating mature OLs and the morphological parameters measured. Diameters of five myelin sheath fibers per newly generated myelinating mature OL were measured at six positions along each myelin internode. (b, c) The average fiber diameter of myelin sheaths per newly generated myelinating mature OL in young and aged mice, measured throughout the corpus callosum (b) or within the anterior, middle, and posterior parts of the corpus callosum (c). (d, e) The individual fiber diameter of myelin sheaths from all newly generated myelinating mature OLs in young and aged mice in the entire corpus callosum (d) or within the anterior, middle, and posterior parts of the corpus callosum (e). N (young,

aged) = (15 cells from five mice, 17 cells from nine mice, b, f), (anterior: five cells, five cells; middle: five cells, seven cells; posterior: five cells, five cells, c), (75 sheaths, 85 sheaths, d), (anterior: 25 sheaths, 25 sheaths; middle: 25 sheaths, 25 sheaths; posterior: 35 sheaths, 25 sheaths, e). Data are presented as the mean ± SEM (b, c) and the median (IQR) (d, e). Student's *t*-test (b, c) and Mann–Whitney *U*-test (d, e).
